# PI3K/AKT/mTOR Signaling Pathway Is Downregulated by Runzaoling (RZL) in Sjögren's Syndrome

**DOI:** 10.1155/2022/7236118

**Published:** 2022-09-12

**Authors:** Ping Zeng, Zong Jiang, Zhaowei Huang, Ying Huang, Hui Xu, Changming Chen, Wukai Ma

**Affiliations:** ^1^Department of Rheumatology and Immunology, The Second Affiliated Hospital, Guizhou University of Traditional Chinese Medicine, Guiyang, China; ^2^The Second Clinical School of Medicine, Guizhou University of Traditional Chinese Medicine, Guiyang, China

## Abstract

Infiltration and aggregation of lymphocytes in exocrine glands are the basic pathological manifestations of Sjögren's syndrome (SS), and the incidence of SS has been increasing year by year in recent years. To explore the potential signaling pathway of Runzaoling (RZL) in alleviating SS, the possible targets of RZL in SS were firstly explored through network pharmacology, and then, the regulation of PI3K/AKT/mTOR signaling in NOD mice and Th17 cells was verified. 75 8-week-old NOD mice were casually classified into 5 groups: model; hydroxychloroquine; high, medium, and low dose RZL groups, with 15 in each; and 15 BALB/c mice were employed as control group. After 10 weeks of continuous intragastric administration in mice and 24 hours of drugs intervention in Th17 cells, histopathology was observed by HE staining, and the gene transcription levels were identified by real-time quantitative PCR (RT-qPCR). The protein expressions were detected by western blotting (WB). The findings showed that high and medium dose RZL group could attenuate the submandibular gland tissue damage. The results indicated that the mRNA expressions of PI3K, AKT, mTOR, STAT3, and IL-17 in SS mice and in IL-17 stimulation of Th17 cells were dramatically increased compared with control group and decreased to varying degrees after RZL intervention. The trend of phosphorylated PI3K/AKT/mTOR and STAT3 and IL-17 protein expression in NOD mice and Th17 cells were consistent with mRNA. RZL can downregulate STAT3 and IL-17 expressions in the submandibular gland of NOD mice and in Th17 cells via regulating the PI3K/AKT/mTOR signaling pathway. Moreover, RZL could reduce the activation of CD4+ T lymphocyte differentiation to Th17 cells.

## 1. Introduction

Sjögren's syndrome (SS), also is familiar with autoimmune exocrine adenopathy, is a diffuse connective tissue disease represented by specific autoantibodies (anti-SSA/SSB) and lymphocyte infiltration. The lacrimal glands and salivary glands were mainly affected by SS and presented as an exocrinopathy. Some patients may be accompanied by multiple systemic involvement of the respiratory system, kidneys, blood system, and nervous system [1]. Epidemiological surveys show that the global prevalence of SS is 0.05% to 1%, and the incidence in China is 0.33% to 0.77%, mainly in women aged 40 to 50, with a male to female ratio of about 1 : 9 [2, 3]. The exact etiology and pathogenesis of SS still remain explored, and most studies believe that it is correlated with immune disorders, genetics, and the environment factors [4–6]. Due to the increasing incidence of SS year by year, the characteristics of long-term, recurrent, and refractory make the treatment difficult. The life-long drug maintenance treatment was required in most patients, provide families with medical expenses and loss of labor, and bring a heavy burden to society.

Runzaoling (RZL), the antidote to remove blood stasis, is a clinically experienced prescription. It is composed of Zhongjiefeng (*Sarcandrae Herba*), Shanyinhua (*Lonicerae hypoglauca*), Liuyuexue (*Serissa Japonica*), Chishao (*Paeonia veitchii Lynch*), Sanqi (*Panax Notoginseng*), Huangjing (*Polygonatum sibiricum F. Delaroche*), Xuanshen (*Scrophularia ningpoensis Hemsl*), and other medicines. Zhongjiefeng, Shanyinhua, and Liuyuexue are antifebrile and antidotal; Xuanshen and Huangjing nourish yin and replenish blood; Chishao and Sanqi facilitate blood circulation and remove blood stasis; all the medicines are effective in clearing away heat and detoxifying, moistening dryness, and promoting blood circulation. Preliminary experimental studies have confirmed that RZL can not only downregulate the serum Th1/Th2 and IL-17 levels and regulate the balance of Th17/Treg cells in SS model mice but also increase the water intake and submandibular gland index of SS mice and improve the submandibular gland lymphocyte infiltration and other pathological changes of model mice [7–10]. Clinical studies have also confirmed that RZL formula has certain advantages in improving clinical symptoms and reducing inflammatory indicators in patients with SS [11, 12].

T helper cell type 17 (Th17) is a CD4+ T cell subset discovered in recent years, which secretes IL-6, IL-17A (IL-17), IL-17F, and TNF-*α*. These cytokines are mattering a lot in autoimmune diseases, chronic inflammatory responses, tumors, and other disease processes [13]. Th17 lymphocytes are crucial in the progress and occurrence of SS, and the cytokines IL-6, IL-1*β*, TNF-*α*, and IL-17 are often overexpressed. Tasaki et al. [14] pointed out that Th17 poses a crucial role in the incidence and progress of pSS (primary Sjögren's syndrome). The IL-17 family contains six members at the minimum including IL-17A (clinically mainly refers to IL-17), IL-17B, IL-17C, IL-17D, IL-17E, and IL-17F [15]. So far, only IL-17A and IL-17F have been found to be related to the occurrence of autoimmune diseases, and the functions of the remaining IL-17 family members are still unclear [16].

On the basis of previous clinical and experimental research, this topic further explored the regulatory mechanism of the detoxification and blood stasis of RZL on SS from the PI3K/AKT/mTOR signaling pathway. RZL, IL-17 stimulators, and IL-17 inhibitors were used to intervene Th17 cells, and the intervention effect of the detoxification and blood stasis drug RZL on Th17 cells and the effect on PI3K/AKT/mTOR signaling pathway were observed. This experiment attempts to explore the possible mechanism and potential therapeutic targets of RZL in treating SS and lay a theoretical and experimental basis for the clinical development and application of RZL.

## 2. Materials and Methods

### 2.1. Network Pharmacology

The Traditional Chinese Medicine Systems Pharmacology Database and Analysis Platform (TCMSP) was exploited to receive components of RZL which were acquired from http://lsp.nwu.edu.cn/tcmsp.php, and putative RZL target genes were collected from SwissTargetPrediction. SS-related gene targets were screened in databases such as Drugbank, Genecards, OMIM, PharmGkb, and TTD by using “Sjögren's syndrome” as a search term. The intersection genes of RZL prediction and SS target gene were obtained by using the SangerBox data analysis platform (http://sangerbox.com/). And the protein-protein interaction (PPI) network map was further constructed to explore the latent mechanism of RZL on SS by exploiting STRING (https://string-db.org/). The drug-disease target network graph was drawn with the usage of Cytoscape 3.6.1 software. After the RZL and SS intersection genes were imported into Cytoscape, gene oncology (GO) biological process analysis was subsequently performed with an adjusted *P* value of < 0.05 by the ClueGO plugin, and the enrichment analysis results were displayed as bubble charts.

### 2.2. Preparation of RZL

RZL formula is composed of 30 g of Zhongjiefeng, 10 g of Shanyinhua, 10 g of Liuyuexue, 10 g of Chishao, 5 g of Sanqi, 15 g of Huangjing, and 10 g of Xuanshen, provided by the Chinese Pharmacy of the Second Affiliated Hospital of Guizhou University of Traditional Chinese Medicine. The above Chinese herbal medicine pieces were soaked in water for 30 min, subsequently, decocted for 30 min and filtered. Then, a small amount of water was added to the medicinal residues and continued to decoct for 10-20 min and filtered. Combining the two filtrates and perform decoction again until the liquid of the crude drug concentration was 1.8 g/mL. The liquid was served as the high dose RZL, the medium dose RZL was obtained by taking part of the high dose RZL liquid and dilute it with the same amount of deionized water to 0.9 g/mL and the low dose RZL to 0.45 g/mL.

### 2.3. SS Model Mice

Seventy-five female nonobese diabetic (NOD) mice aging 8 weeks were casually classified (5 groups, 15 mice in each group), and 15 BALB/c mice of the same age were employed as the control group. The specific groups are as follows: (1) blank control group (gavage of 0.2 mL of deionized water every day); (2) model group (gavage of 0.2 mL of deionized water every day); (3) hydroxychloroquine group (gavage of 0.2 mL of hydroxychloroquine suspension every day, 4 mg/mL); (4) high dose RZL group (18 g·kg^−1^/d); (5) medium dose RZL group (9 g·kg^−1^/d); (6) low dose RZL group (4.5 g·kg^−1^/d).

### 2.4. Collection and Processing of Experimental Specimens

After 10 weeks of gavage treatment, the mice were anesthetized by intraperitoneal injection; the limbs were fixed after weighing; the abdomen was disinfected by spraying alcohol; the abdominal skin was cut to open the abdominal cavity; the abdominal organs, tissues, and ligaments were separated; the abdominal aorta was exposed; and blood is drawn with a lancet. The bilateral submandibular glands were excised from the mandible. Most of the submandibular glands were quickly placed in liquid nitrogen and then transmitted to a -80°C low-temperature refrigerator for cryopreservation for the detection of various indicators.

### 2.5. HE Staining

Tissue was fixed in 4% paraformaldehyde, and after 48 h, rinsed off with running water, dehydrated in 70%, 80%, and 90% ethanol step by step for 5 min each, dehydrated in absolute ethanol for 10 min, and then permeabilized with xylene. The tissue was embedded in paraffin at 65°C, sliced with a microtome, with a thickness of 4 *μ*m, and dried in an incubator at 45°C. The xylene was dewaxed twice for 10 min each, then soaked in anhydrous ethanol, 95%, 90%, 50%, 70%, and 80% ethanol for 10 min each, and washed with distilled water. Hematoxylin staining was performed for 5 min, followed by eosin staining for 30 s, rinsed with distilled water, dehydrated with alcohol gradient for 10 s each, mounted with resin glue, and observed under an optical microscope (Olympus FSX100; Olympus Corporation).

### 2.6. qPCR

The submandibular gland stored in the -80°C freezer was quickly taken out, add liquid nitrogen to grind, break the cells, and collect in an EP tube; afterward, each 50-100 mg of tissue was lysed with 1 mL of Trizol reagent, and let stand for 10 minutes at room temperature. The lysed tissue was washed with 0.2 mL of chloroform, 0.5 mL of isopropanol, and 1 mL of 75% ethanol in sequence to precipitate RNA, and naturally dried at room temperature. After the ethanol in the EP tube has completely evaporated, dissolve the RNA precipitate with RNase-free water. Pipette 2 *μ*L of RNA sample, mix and drop it on a microquartz detection plate, and use a microplate reader to detect its OD value to evaluate RNA purity and calculate RNA concentration. Reverse transcription was performed at 37°C for 15 min, 85°C for 5 s, and 4°C for 10 min for cDNA synthesizing. TB Green Premix Ex Taq II is used for real-time PCR fluorescence quantification, and the PCR reaction program is started according to different reaction temperatures and times of denaturation, annealing, and extension. A fluorescence quantitative PCR instrument (BIO-RAD, USA) was used to record the Ct value of each sample, and the 2^-△△CT^ method was used for a relative quantitative analysis. The PI3K, AKT, mTOR, IL-17A, and STAT3 primer sequences were acquired from the NCBI GenBank database, and the primer sequences are shown in Table 1.

### 2.7. Western Blotting

The submandibular glands of mice were cut into small pieces with tissue scissors, and a proper volume of RIPA tissue lysis buffer addition was done. Cryogenic grinding was performed on ice with a glass homogenizer until the glandular tissue was fully lysed. Transfer the grinding liquid to a 1.5 mL EP tube. Centrifuge at 4°C, 14000 rpm for 5 min, and the total protein of mouse submandibular gland tissue was obtained by aspirating the supernatant. Based on the instructions of the BCA Protein Concentration Assay Kit (Solarbio, China), the protein concentration was determined. The samples and 5 × SDS loading buffer were prepared with a proportion of 4 : 1 to prepare a protein mixture, which was then experiencing a 5 min 100°C water bath to denature the protein. The electrophoresis was started in a constant voltage of 150 V. The PDVF membrane was cut to an appropriate size and activated by soaking in methanol for 5 min. The protein was transferred to the membrane in a constant voltage of 25 V lasted for 4-7 min. After transference, place the protein-containing side up, wash with TBST for 1 min, and block with 5% skim milk for 2-3 h. The PVDF membrane was completely immersed in corresponding primary antibody and incubated at 4°C. The primary antibody used was as follows: IL-17 (#A19742, 1 : 500, abclonal, Wuhan China), p-PI3K (#4228 T, 1 : 1000, CST, USA), p-AKT (#4060 T, 1 : 1000, CST, USA), p-mTOR (#AP0115, 1 : 500, abclonal, Wuhan China), STAT3 (#A1192, 1 : 500, abclonal, Wuhan China), and *β*-actin (#AC026, 1 : 500, abclonal, Wuhan China). The next day, PVDF membrane was rinsed off using TBST for 7 min and repeated three times. After the addition of the diluted secondary antibody, the membrane was incubated at room temperature (RT) for 1.5 h on a shaker. The membrane was washed with TBST for 7 min and repeated three times. The chemiluminescence imaging system (ChemiDoc Touch Imaging System, BIO-RAD) was used for membrane exposed and developed, the developed image was saved, and the gray value of the image was calculated by Image J software.

### 2.8. Immunohistochemistry

MaxVision™ technology (Maixin Bio, China) was used for immunohistochemical (IHC) analysis. The submandibular gland tissue was removed from the -80°C freezer and fixed with 4% paraformaldehyde (Solarbio, Shanghai, China). The tissue was dehydrated, embedded using paraffin, and then sectioned into 5 μm slicer. The slicer was dried at 60°C for 1 h, followed by dewaxing with xylene and alcohol, and immersed in Tris-EDTA antigen retrieval buffer and boiled for 15 minutes. The section was blocked with 5% goat serum at RT for 1 h, 3% hydrogen peroxide was added dropwise and protected from light at RT for 30 minutes. The section was incubated with primary antibody at 4°C, and the next day incubated with the HRP-polymer-conjugated antibody at 37°C for 2 h. Diaminobenzidine was used for color development for 3 min, and nucleus were stained using hematoxylin (Servicebio, Wuhan, China). After drying, the slides were mounted with neutral gum, and image acquisition was performed with inverted microscope (Olympus, Japan).

### 2.9. Th17 Cell Induction

Peripheral venous blood was drawn from SS patients with heparin anticoagulated vacuum blood collection tubes. The density gradient centrifugation was used for peripheral blood mononuclear cells (PBMCs) preparation. One day before the induction of Th17 cells, a PBS solution containing 5 *μ*g/mL Anti-Human CD 28 (AH028, Multi Sciences, China) and Anti-Human CD 3 (AH003, Multi Sciences, China) was supplemented for a Petri dish coating, and condition was set at 4°C for 24 h. The next day, the isolated PBMCs were added to precoated dishes. At the same time, 10 ng/mL IL-6, 10 ng/mL IL-1*β*, 1 ng/mL TNF-*α*, and 1 ng/mL TGF-*β* were added to induce differentiation of Th17 cells for 72 h.

Cells were incubated in RPMI 1640 medium (BasalMedia, China) containing 10% FBS (Life-iLab, China) and 1% penicillin-streptomycin (Beyotime, China). Six groups were classified in cell experiments: blank control group, IL-17 stimulation group (2.5 *μ*M, SR0987), IL-17 blockade group (1 *μ*M, GSK2981278), high dose RZL group (2.5 mg/mL), medium dose RZL group (1.5 mg/mL), low dose RZL group (1 mg/mL). A 37°C, 5% CO2 constant temperature incubator was used for drug intervention for 24 h. The cell supernatants of the above groups were dispensed into labeled 2 mL EP tubes, and sample storage was performed at -80°C refrigerator for subsequent detection of relevant indicators.

### 2.10. Statistical Analysis

The experimental data and graphs of each group were analyzed using GraphPad Prism 8 software, and the measurement data were expressed as mean ± standard deviation (^−^*x* ± *s*). All data were analyzed using *t*-test or One-Way ANOVA. Each group of experiments was repeated three times or more, and *P* < 0.05 indicated that the analysis results were statistically significant.

## 3. Results and Discussion

### 3.1. The Potential Targets of RZL in Regulating SS

**Figure 1:**
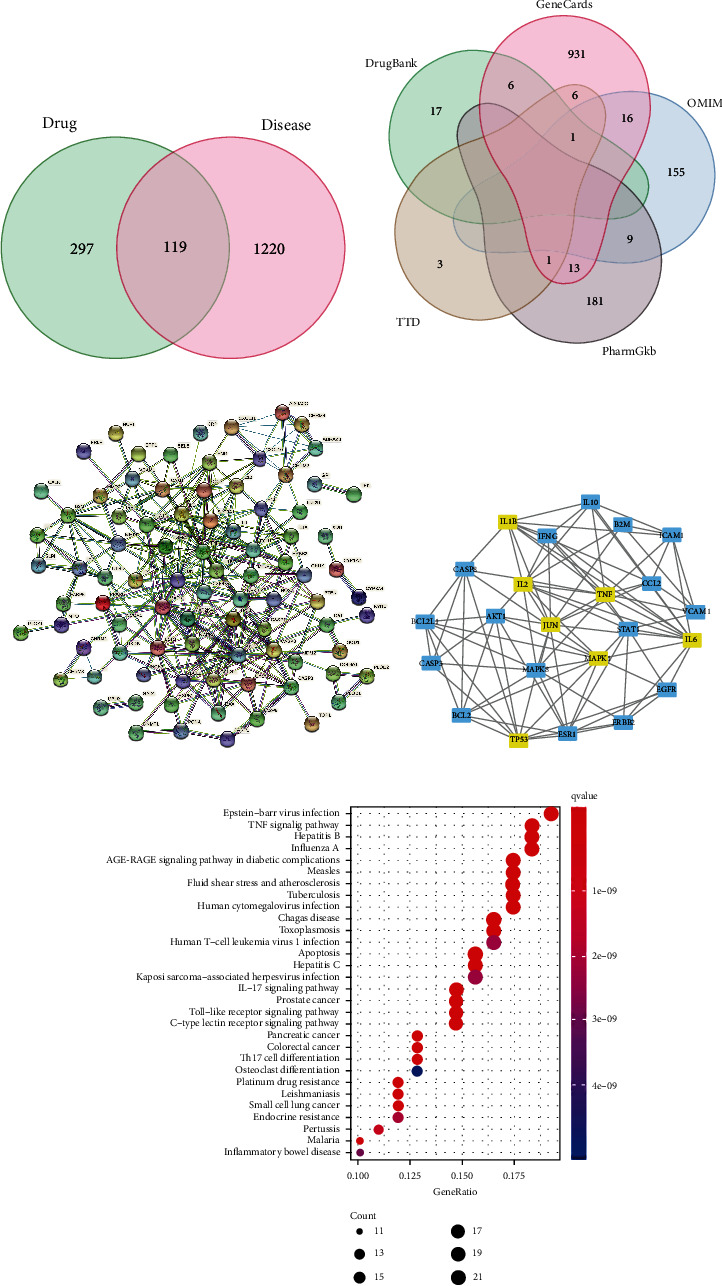
RZL can regulate SS through the AKT/mTOR pathway. (a) Venn diagram of RZL and SS targets. (b) Venn diagram of the source of SS disease targets. (c) (String) and (d) (Cytoscape) PPI plots of disease and drug intersection targets. (e) Bubble plot of GO biological process enrichment.

**Figure 2:**
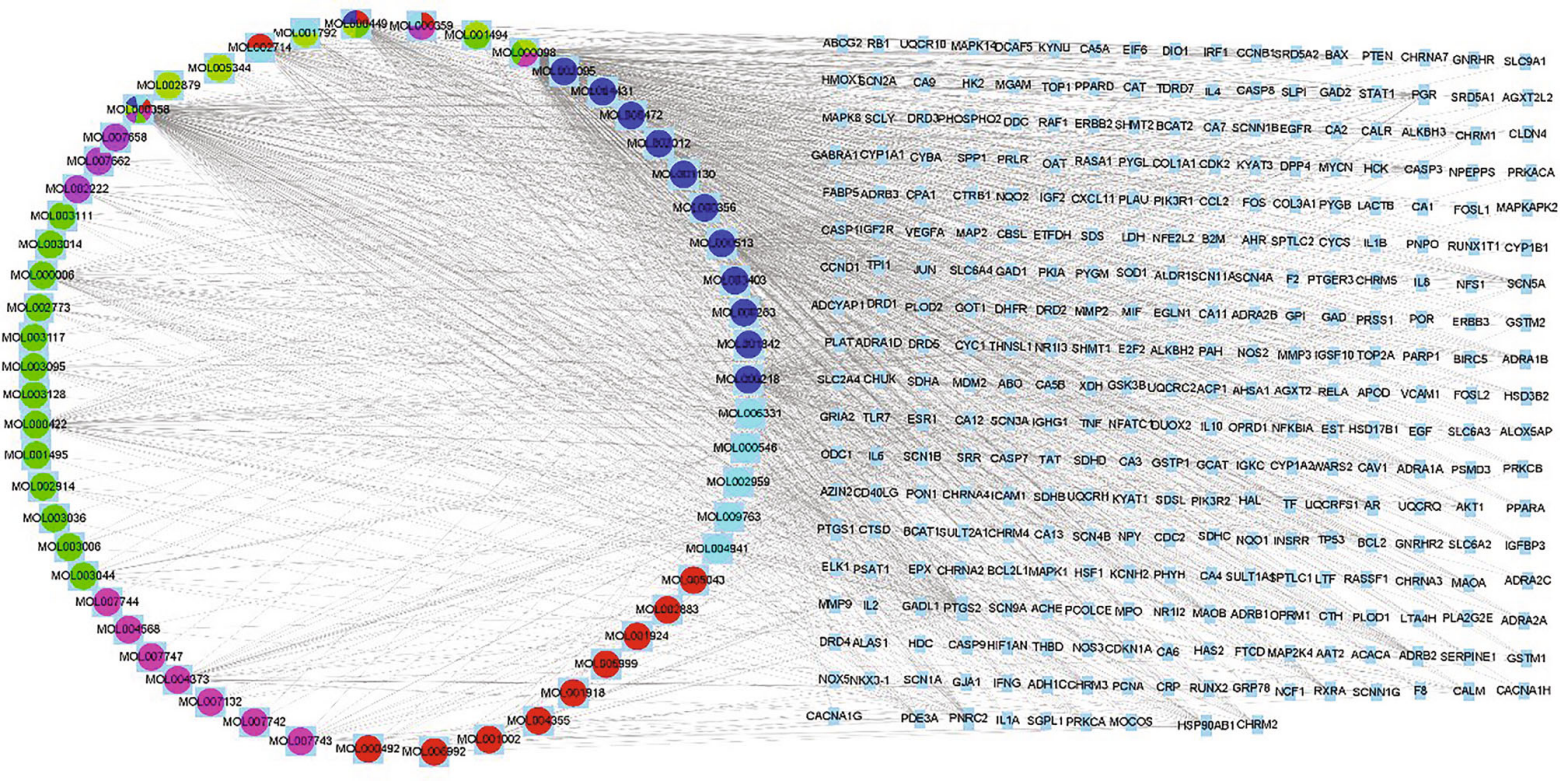
Network diagram of RZL components and SS disease targets. The magenta circles represent the ingredients of Zhongjiefeng, green circles for Shanyinhua, blue circles for Liuyuexue, red circles for Chishao, chartreuse circles for Sanqi, cyan-blue circles for Huangjing, and purple circles for Xuanshen.

The intersection genes were searched through all collected RZL drug component targets gene and SS disease targets gene, and a Venn diagram was drawn (Figure 1(a)). A total of 416 drug targets and 1339 disease target genes were retrieved. RZL and SS shared 119 common targets. The source of the 1339 SS target genes is shown in Figure 1(b). PPI network mapping of 119 intersecting target genes was performed through String and Cytoscape websites (Figures 1(c) and 1(d)). The results illustrated that AKT1, mTOR, IL-6, and IL-17 have multiple edges connected to nodes in the PPI network graph and thus are reasonable to be the key targets of RZL in treating SS. GO biological process enrichment analysis of the obtained intersection genes illustrated that IL-17 signaling pathway and Th17 cell differentiation may occupy a significant position in the process of RZL treatment of SS (Figure 1(e)). In addition, RZL components and SS disease target networks were mapped using Cytoscape (Figure 2).

### 3.2. RZL Inhibits Inflammatory Reactions in the Submandibular Gland of NOD Mice

**Figure 3:**
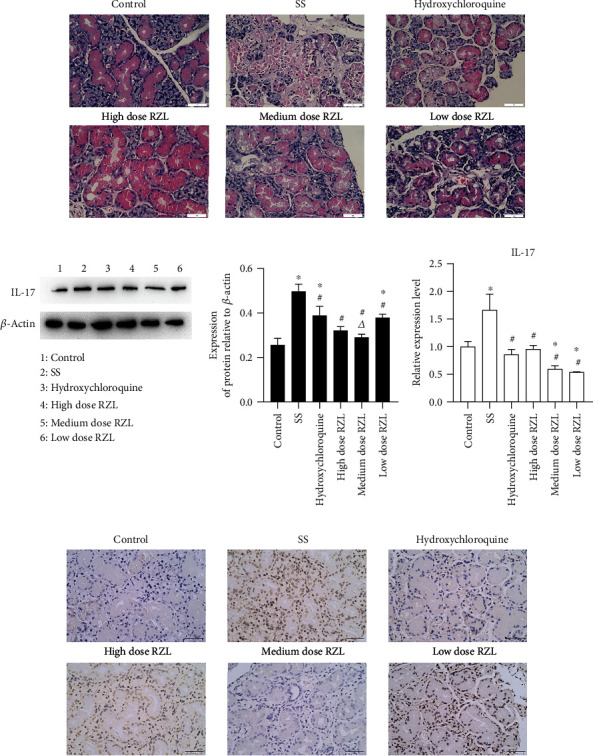
RZL inhibits inflammatory reactions in the submandibular gland of SS mice. (a) HE staining of submandibular gland tissue after different treatment. Scar bar = 50 *μ*m (400×). (b) Representative immunoblot bands of IL-17 and quantitative expression of IL-17 protein in mouse submandibular gland tissue. (c) IL-17 mRNA expression in mouse submandibular gland tissue. (d) Immunohistochemistry was used for IL-17 protein determination. Scar bar = 50 *μ*m (400×). In comparison to the control group, ^∗^*P* < 0.05; in comparison to the model group, ^#^*P* < 0.05; in comparison to the hydroxychloroquine group, *^Δ^P* < 0.05.

The histopathology of the submandibular gland tissue of mice was observed by HE staining. As shown in Figure 3(a), the size of the acinus in the control group was basically uniform, and the structure of the acinus and duct was normal. In comparison to the control group, the acinar cells in the model were significantly reduced and varied in size, with moderate to severe lymphocytic infiltration in the interstitium, some ducts fused and expanded, and local tissue degeneration and necrosis. Mild lymphocyte infiltration was found in the hydroxychloroquine group and the high and medium dose RZL group; moreover, some ducts were dilated in them, the size of the acinus was different, and a small number of acinar and ductal structures was destroyed. In comparison to the hydroxychloroquine group, the lymphocyte infiltration in high dose RZL group was markedly improved.

In addition, we used WB, qPCR, and immunohistochemistry to detect the expression of inflammatory factor IL-17 in submandibular gland tissue, then further explore the inflammatory reaction of submandibular gland tissue after RZL treatment (Figures 3(b)–3(d)). The mRNA levels of IL-17 in the submandibular gland of the mice in SS mice were clearly enhanced as compared to control (*P* < 0.05). After RZL treatment, the IL-17 mRNA expressions were decreased in hydroxychloroquine and RZL-treated mice as compared to the model mice. The medium dose RZL group could downregulate IL-17 mRNA comparing with the hydroxychloroquine group (Figure 3(c)). The results demonstrated that compared with the control, IL-17 protein expressions in the submandibular glands of the model mice were increased. The protein expression of each RZL-administered in different doses was decreased to different degrees as compared to the model group. Overall, the protein expression of IL-17 in the medium dose RZL group was reduced in contrast to the hydroxychloroquine group, suggesting that the efficacy of medium dose RZL was stronger than that of the hydroxychloroquine group (Figures 3(b) and 3(d)).

### 3.3. RZL Downregulates PI3K/AKT/mTOR Pathway in the Submandibular Glands of NOD Mice

**Figure 4:**
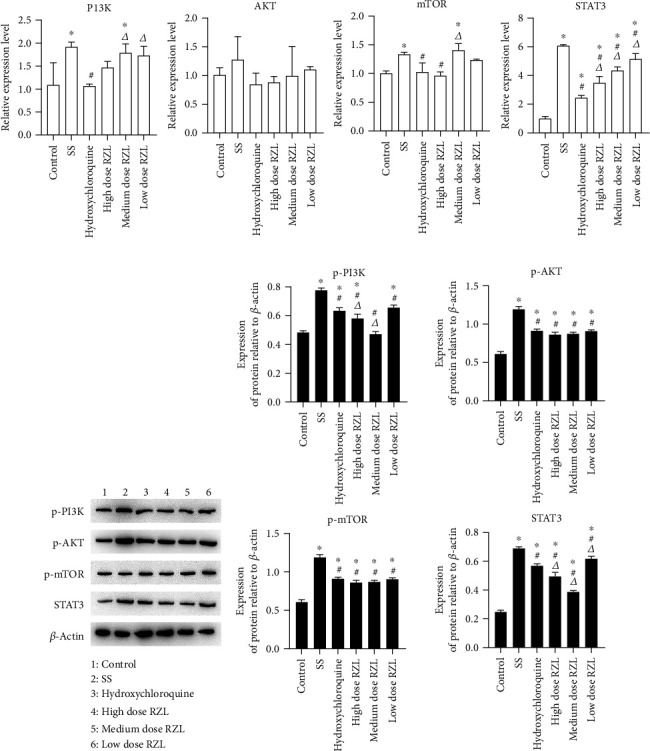
RZL regulates PI3K/AKT/mTOR pathway expression in SS mice. (a) Quantitative expression of PI3K, AKT, mTOR, and STAT3 mRNA in mouse submandibular gland tissue. (b) Representative immunoblot bands. (c) Expression of p-PI3K, p-AKT, p-mTOR, and STAT3 proteins in mouse submandibular gland tissue. In comparison to the control group, ^∗^*P* < 0.05; in comparison to the model group, ^#^*P* < 0.05; in comparison to the hydroxychloroquine group, *^Δ^P* < 0.05.

We found that RZL's intervention in SS disease process may be related to AKT and mTOR through network pharmacology experiments. With the aim to verify the possible regulatory role of AKT and mTOR in RZL treatment of SS, we used spontaneous SS model NOD mouse to verify the PI3K/AKT/mTOR pathway expressions in mouse submandibular gland tissue by qPCR and WB after RZL treatment. The mRNAs of PI3K, AKT, mTOR, and STAT3 in the submandibular gland of the SS mice were clearly increased as compared to control, and there was no significant difference in AKT. After the treatment of RZL, the mRNA levels were decreased to varying degrees in contrast to model group. The high and medium dose RZL group could upregulate PI3K, AKT, and STAT3 mRNA in comparison with the hydroxychloroquine group (Figure 4(a)).

The results demonstrated that the protein expressions of p-PI3K, p-AKT, p-mTOR, and STAT3 in submandibular glands of SS mice were markedly increased in contrast to control (*P* < 0.05). Comparing with SS mice, the protein expressions of each RZL-administered mice in different doses decreased to different degrees (*P* < 0.05). The protein expressions of p-PI3K, p-AKT, p-mTOR, and STAT3 in the high and medium dose RZL group were reduced in contrast to the hydroxychloroquine group, and there was statistical significance in p-PI3K and STAT3 expressions, suggesting that the efficacy of was stronger than that of the hydroxychloroquine group (Figures 4(b) and 4(c)).

### 3.4. RZL Downregulates the mRNA Level of PI3K/AKT/mTOR Pathway in Th17 Cells

**Figure 5:**
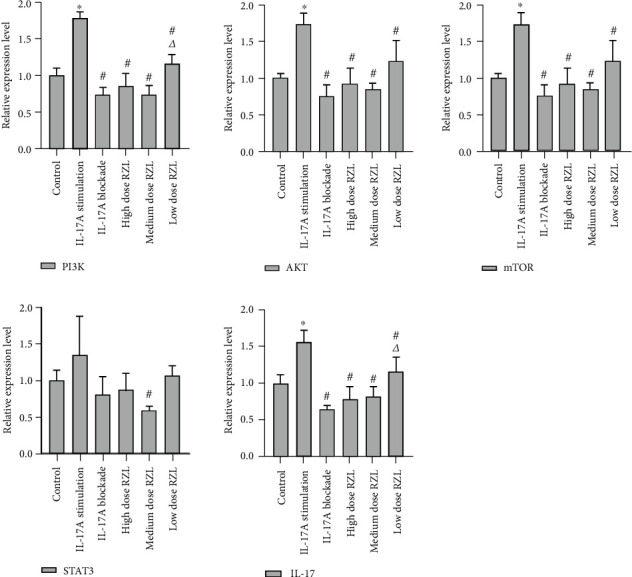
RZL regulates gene expression of PI3K/AKT/mTOR pathway in Th17 cells. Relative expression of PI3K, AKT, mTOR, STAT3, and IL-17 mRNA in Th7 cells. ^∗^*P* < 0.05 as compared to control; ^#^*P* < 0.05 as compared to IL-17A stimulation; *^Δ^P* < 0.05 as compared to IL-17A blockade.

In addition to detect the PI3K/AKT/mTOR pathway regulatory function by RZL in the submandibular gland tissue of SS mice, we also induced and cultured Th17 cells and verified the regulation of RZL on the PI3K/AKT/mTOR pathway at cellular level. The regulatory effect of RZL on the mRNA level of PI3K/AKT/mTOR pathway in Th17 cells was verified by qPCR. As illustrated in Figure 5, in comparison to the control, PI3K, AKT, mTOR, and IL-17 mRNA expressions were significantly upregulated in the IL-17A stimulated group (*P* < 0.05), and the expression of STAT3 mRNA was also upregulated in the IL-17A-stimulated group (*P* > 0.05). The mRNA levels of PI3K, AKT, mTOR, STAT3, and IL-17 in the IL-17A blocking group were lower in contrast to those in control. To make a comparison of the control and RZL-treated group, after RZL administration, the mRNA expressions of PI3K, AKT, mTOR, STAT3, and IL-17 were downregulated in different degrees, and the downregulation effect was better in high dose RZL group.

### 3.5. RZL Downregulates the Phosphorylated Protein Expression of PI3K/AKT/mTOR Pathway in Th17 Cells

**Figure 6:**
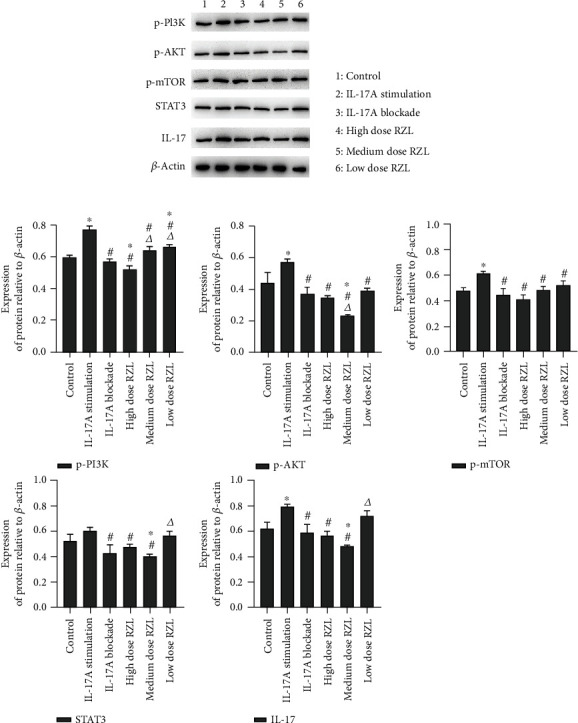
RZL regulates the of PI3K/AKT/mTOR pathway protein expressions in Th17 cells. (a) Representative immunoblot bands for proteins. (b) Relative expression of p-PI3K, p-AKT, p-mTOR, STAT3, and IL-17 proteins in Th7 cells. ^∗^*P* < 0.05 as compared to control; ^#^*P* < 0.05 as compared to IL-17A stimulation; *^Δ^P* < 0.05 as compared to IL-17A blockade.

In comparison to control, p-PI3K, p-AKT, p-mTOR, and IL-17 proteins in IL-17A stimulation group were significantly upregulated (*P* < 0.05). STAT3 expression was upregulated in the IL-17 stimulation group (*P* > 0.05). The proteins of p-PI3K, p-AKT, p-mTOR, STAT3, and IL-17 in the IL-17A blockade group were downregulated as compared to control. In contrast to control group, the protein expressions of p-PI3K, p-AKT, p-mTOR, STAT3, and IL-17 were all downregulated to varying degrees after high dose RZL administration (Figure 6).

## 4. Discussion

Although the pathogenesis of systemic autoimmune disease SS has not been completely clarified, the infiltration, proliferation, and foci of lymphocytes in exocrine glands are the basic pathological manifestations of SS. T and B lymphocytes and autoantibodies in vivo were activated abnormally whose generation is a central principle for the occurrence and persistence of autoimmune inflammation in exocrine glands [17, 18]. The latest research shows that involvement of Th17 cells is crucial in incidence of SS. The increased differentiation of Th17 in SS patients results in the massive secretion of IL-17. Activated IL-17 is a strong initiating factor of inflammation which poses a function in inducing chronic tissue damage in exocrine glands [19, 20].

After activation, IL-17A not only exerts its own proinflammatory effect but also further induces the generation of other potential proinflammatory factors. Studies have proved the raising number of IL-17-producing cells in salivary glands of SS patients, and infiltration has been observed in them, with Th17 cells being the major infiltrating cell subset [21]. In addition, IL-17 levels and Th17 cells in peripheral blood and ocular surface were also elevated in SS patients in contrast to healthy subjects [22, 23] and IL-17 levels in tears relevant to tear film breakup time and filter paper test, indicating that IL-17 was crucial in the incidence of SS [24, 25]. The above findings were also validated in the SS mouse model, which proves that the amount of IL-17 and Th17 cells were increased in the salivary and lacrimal glands of SS mice [26, 27]. The regulating mechanisms of Th17 cell differentiation have been extensively explored and, in addition to inflammatory cytokines like IL-6, TGF-*β*, and IL-23, also include numerous intracellular signaling cascades. Among them, the PI3K/AKT/mTOR signaling is a more complete pathways related to cells. This pathway can regulate life processes such as cell proliferation, metabolism, differentiation, and survival, and it is abnormally expressed in a variety of autoimmune diseases [28–30]. Studies have proved [31–33] that PI3K/AKT/mTOR signaling has a positive regulatory effect on the differentiation of Th17 cells. Activation of mTOR induces phosphorylation of STAT3 that is essential in Th17 differentiating and production of related cytokines such as IL-17A, IL-17F, IL-21, and IL-22.

Our experiments demonstrated that RZL could reduce p-PI3K, p-AKT, p-mTOR, IL-17A, and STAT3 proteins and PI3K, AKT, mTOR, IL-17A, and STAT3 mRNA levels in submandibular gland tissue of NOD mice. Studies have reported that [34] SS patients have increased Th17/IL-17-related factors and their mRNAs in local saliva, salivary glands, tears, lacrimal glands, or systemic peripheral blood. After mTOR activation, it can positively modulate Th17 cell differentiating, implement histone modification of the IL-17 gene by mediating STAT3 phosphorylation, aggravate the generation of IL-17, and aggravate the inflammatory response. At the same time, the persistence of inflammation will lead to continuously upregulated autophagy level in glandular cells and autophagic programmed death and apoptosis occur. Deficiency of the mTOR gene or the use of mTOR inhibitors results in a marked reduction in the differentiation of Th17 cells [35]. RZL may limit the progression of SS by suppressing the Th17 cell differentiation in patients with SS and reducing the level of IL-17-related cytokines in patients, thereby achieving the purpose of treating SS. The Th17 cell experiments verified the function of RZL in regulating the expression of PI3K/AKT/mTOR pathway proteins was consistent with that in animal experiments. In the Th17 cell experiment, an IL-17 stimulation group and a blockade group were also added. In the IL-17 blockade group, phosphorylated PI3K/AKT/mTOR, STAT3, and IL-17 protein and mRNA genes were downregulated. Activated mTORC1 can histone-modify the IL-17 gene by promoting STAT3 phosphorylation, thereby promoting the production of IL-17, aggravating the inflammatory response, and promoting the SS process. RZL can treat SS by downregulating the PI3K/AKT/mTOR signaling pathway, inhibiting the differentiation of Th17 cells, and reducing the level of IL-17 production; and the inflammatory microenvironment in the exocrine glands improved.

## 5. Conclusions

RZL can inhibit the phosphorylated PI3K/AKT/mTOR protein expressions in NOD mice and Th17 cells, thereby inhibiting the production of STAT and IL-17 and affecting the differentiation of Th17. This pathway of PI3K/AKT/mTOR may be related to RZL improving the inflammatory microenvironment and relieving SS symptoms and help provide usable insights for probing the mechanism of RZL in SS and SS treatment.

## Figures and Tables

**Table 1:** Primer sequences used for gene transcription levels determination.

Primers	Sequences (5′-3′)
PI3K-F	CGAGACGGCACTTTCCTTGT
PI3K-R	CGGTGGCAGTCTTGTTAATGAC
Akt-F	GCCGCCTGATCAAGTTCTCC
Akt-R	GGCTTCTGGACTCGGCAATG
mTOR-F	CAGTTCGCCAGTGGACTGAAG
mTOR-R	GCTGGTCATAGAAGCGAGTAGAC
IL-17A-F	TCAATGCGGAGGGAAAGCTG
IL-17A-R	CCACCAGCATCTTCTCGACC
STAT3-F	CACCTTGGATTGAGAGTCAAGAC
STAT3-R	AGGAATCGGCTATATTGCTGGT
GAPDH-F	AGGTCGGTGTGAACGGATTTG
GAPDH-R	TGTAGACCATGTAGTTGAGGTCA

## Data Availability

The data supporting the findings of this study are available from the corresponding author upon request.
